# Genetic and phenotypic landscape of pediatric-onset epilepsy in 142 Indian families: Counseling and therapeutic implications

**DOI:** 10.1111/cge.14495

**Published:** 2024-02-19

**Authors:** Purvi Majethia, Namanpreet Kaur, Selinda Mascarenhas, Lakshmi Priya Rao, Shruti Pande, Dhanya Lakshmi Narayanan, Vivekananda Bhat, Shalini S. Nayak, Karthik Vijay Nair, Adarsh Pooradan Prasannakumar, Ankur Chaurasia, Bhagesh Hunakunti, Nalesh Jadhav, Sheeba Farooqui, Mayuri Yeole, Vishaka Kothiwale, Rohit Naik, Veena Bhat, Shrikiran Aroor, Leslie Lewis, Jayashree Purkayastha, Y. Ramesh Bhat, B. K. Praveen, B. L. Yatheesha, Siddaramappa J. Patil, Sheela Nampoothiri, Nutan Kamath, Shahyan Siddiqui, Stephanie Bielas, Katta Mohan Girisha, Suvasini Sharma, Anju Shukla

**Affiliations:** 1 Department of Medical Genetics, Kasturba Medical College, Manipal, Manipal Academy of Higher Education, Manipal, India; 2 Division of Evolution, Infection and Genomics, School of Biological Sciences, Faculty of Biology, Medicine and Health, University of Manchester, Manchester, UK; 3 Department of Paediatrics, Kasturba Medical College, Manipal, Manipal Academy of Higher Education, Manipal, India; 4 Department of Pediatrics, Father Muller Medical College Hospital, Mangalore, India; 5 Paediatric neurology, Dheemahi Child Neurology and Development Center, Shimoga, India; 6 Division of Medical Genetics, Narayana Hrudayalaya Hospitals/Mazumdar-Shaw Medical Center, Bangalore, India; 7 Department of Pediatric Genetics, Amrita Institute of Medical Sciences & Research Centre, Cochin, India; 8 Department of Paediatrics, Kasturba Medical College, Mangalore, Manipal Academy of Higher Education, Manipal, India; 9 Department of Neuro and Vascular Interventional Radiology, Yashoda Hospitals, Hyderabad, India; 10 Department of Human Genetics, University of Michigan Medical School, Ann Arbor, Michigan, USA; 11 Suma Genomics Private Limited, Manipal Center for Biotherapeutics Research, Manipal Academy of Higher Education, Manipal, India; 12 Department of Genetics, College of Medicine & Health Sciences, Sultan Qaboos University, Muscat, Oman; 13 Neurology Division, Department of Pediatrics, Lady Hardinge Medical College and Associated Kalawati Saran Children’s Hospital, New Delhi, India

**Keywords:** epilepsy, genetic testing, genetics, India, therapeutic implications

## Abstract

The application of genomic technologies has led to unraveling of the complex genetic landscape of disorders of epilepsy, gaining insights into their underlying disease mechanisms, aiding precision medicine, and providing informed genetic counseling. We herein present the phenotypic and genotypic insights from 142 Indian families with epilepsy with or without comorbidities. Based on the electroclinical findings, epilepsy syndrome diagnosis could be made in 44% (63/142) of the families adopting the latest proposal for the classification by the ILAE task force (2022). Of these, 95% (60/63) of the families exhibited syndromes with developmental epileptic encephalopathy or progressive neurological deterioration. A definitive molecular diagnosis was achieved in 74 of 142 (52%) families. Infantile-onset epilepsy was noted in 81% of these families (61/74). Fifty-five monogenic, four chromosomal, and one imprinting disorder were identified in 74 families. The genetic variants included 65 (96%) single-nucleotide variants/small insertion-deletions, 1 (2%) copy-number variant, and 1 (2%) triplet-repeat expansion in 53 epilepsy-associated genes causing monogenic disorders. Of these, 35 (52%) variants were novel. Therapeutic implications were noted in 51% of families (38/74) with definitive diagnosis. Forty-one out of 66 families with monogenic disorders exhibited autosomal recessive and inherited autosomal dominant disorders with high risk of recurrence.

## 1 Introduction

Epilepsy is one of the most common neurological disorders which can be present in isolation or can be associated with other comorbidities such as global developmental delay (GDD), intellectual disability (ID), autism spectrum disorder (ASD), and/or behavioral abnormalities. The etiology of epilepsy is heterogeneous and can be attributed to genetic, structural, metabolic, immune, infectious, or idiopathic causes.^1^ It is estimated that 70%–80% of the epilepsies can be genetic, encompassing monogenic and chromosomal disorders arising from rare or ultrarare variants, post-zygotic somatic variants, epigenetic modifications, or multifactorial disorders characterized by a complex genetic architecture.^2–4^

With the advent of next-generation sequencing techniques, a plethora of epilepsy-associated genes and disease-causing variants have been identified in association with epileptic disorders. High genotypic and phenotypic heterogeneity observed among these epileptic disorders substantiates the use of exome or genome sequencing (ES/GS) as a first-tier genetic test in establishing a definitive molecular diagnosis.^5,6^ The utility of genetic tests has been demonstrated in several epilepsy cohort studies where a monogenic cause has been identified in up to 40%–60%, and chromosomal in 7%–10% of individuals with epilepsies associated with other comorbidities.^7–11^ Identification of precise genetic etiology further facilitates tailored precision medicine, and targeted genetic counseling including prognosis and recurrence risk.

Numerous studies have highlighted the significance of elucidating the genetic basis of epilepsy across diverse populations. Large-scale cohort studies play a pivotal role in understanding the diverse phenotypic and genotypic landscape of epilepsies, offering crucial insights into the effectiveness of genomic tests, especially in resource-limited settings. The distinct genetic makeup, environmental factors, possible genetic variations unique to the population, and resource constraints in India emphasize the need for a comprehensive study to assess the genetic burden of epilepsy. We hereby present the phenotypic and genotypic spectrum of epilepsies from 142 Indian families with an emphasis on the utility of sequential genomic testing for achieving genetic diagnosis, selecting optimal treatment, and assisting genetic counseling.

## 2 Methodology

### 2.1 Subject recruitment

We ascertained and recruited individuals presenting with epilepsy with/without comorbidities from October 2019 till June 2023 as a part of an ongoing study. The affected individuals recruited for the study were inpatients as well as outpatients from either genetic or pediatric or pediatric neurology clinics. The diagnosis of epilepsy was based on the current definition of International League Against Epilepsy (ILAE).^1^ Individuals with epilepsy due to acquired causes (stroke, trauma, tumors, neonatal hypoxia, infections) were excluded from the study. The available clinical and electroencephalogram (EEG) data were reviewed by a pediatric epileptologist, and epilepsy syndrome classification was done adopting the latest proposal for the classification of epilepsy syndrome by the ILAE task force on Nosology and Definitions (2022).^12–16^ Informed consents for genetic testing and publication of data were obtained from the families. The informed consents were approved by the institutional ethics committee, Kasturba Medical College, and Kasturba Hospital, India as per the declaration of Helsinki.

### 2.2 Genetic testing

**Figure 1:**
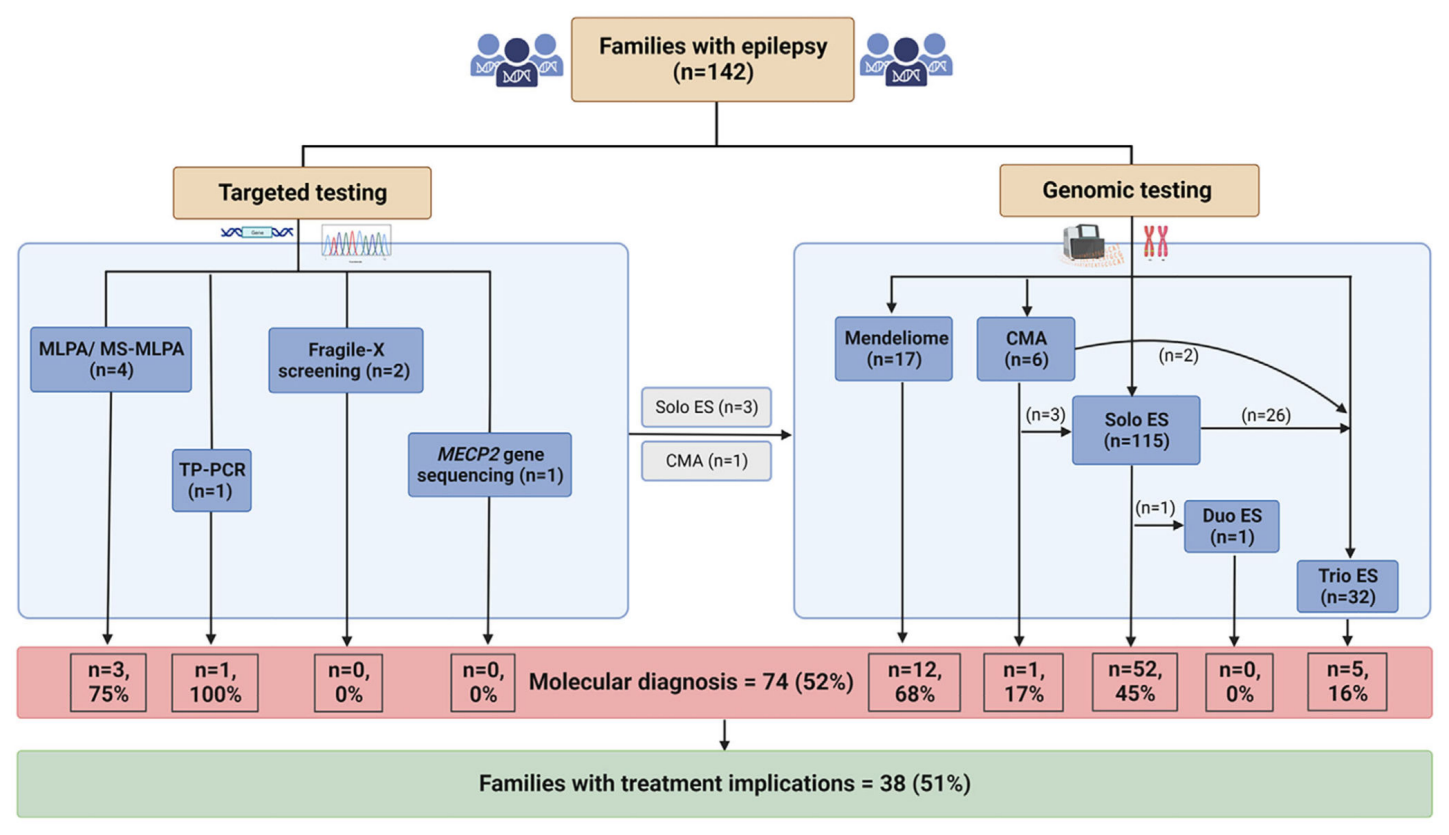
Flowchart depicting the genetic testing strategy employed to achieve molecular diagnosis in 142 families with epilepsies. CMA, chromosomal microarray; ES, exome sequencing; MLPA, multiplex ligation-dependent probe amplification; MS-MLPA, methylation-specific MLPA; TP-PCR, triplet repeat primed PCR. [Colour figure can be viewed at wileyonlinelibrary.com]

Genomic DNA was extracted from the peripheral blood sample of the proband, parents and siblings (as required) using the QIAamp DNA Blood Mini Kit (QIAGEN, Valencia, CA; cat # 51106). The testing strategy included either an exome first or a sequential testing approach in which targeted tests such as fragile-X screening, TP-PCR, Methylation-specific MLPA (MS-MLPA), targeted gene Sanger sequencing or chromosomal microarray (CMA) was followed by ES for the affected individuals based on the clinical phenotype (Figure 1). The disease-causing variants identified in the affected individuals were further validated and segregated in the families using Sanger sequencing. Copy-number variant (CNV) analysis from exome data was performed for individuals in whom no clinically relevant single-nucleotide variants (SNVs) or insertion/deletions (indels) were detected on ES. The CNVs identified using ES data were further validated using CMA, or MS-MLPA. The detailed description of the genetic tests employed along with description of CNV and ES analysis is provided in Data S1.

### 2.3 Therapeutic implications and impact on genetic counseling

After achieving molecular diagnosis, literature review was done to ascertain if any anti-epileptic drugs (AED) and/or any specific therapy is recommended or contraindicated. The following search terms were used in PubMed to find the publications: “seizure + gene name + treatment,” “epilepsy + gene name + treatment,” and “specific gene name + treatment.” The evidence was classified as “strong” when treatment guidelines recommended the use of a particular AED or treatment, as “emerging” when multiple publications supported its use and as “sparse” when benefit was shown only in a single report.

The impact of definitive diagnosis on genetic counseling was assessed by analyzing its influence on recurrence risk and identifying the number of families that could benefit from targeted prenatal diagnosis or preimplantation genetic diagnosis and thus prevention of untreatable disorders.

## 3 Results

We ascertained a total of 161 affected individuals from 152 unrelated families with epilepsy with or without any comorbidities. Of these, 12 affected individuals from 10 unrelated families with findings of novel disease-gene associations have been published earlier and are provided in Table S3.^17–26^

**Figure 2:**
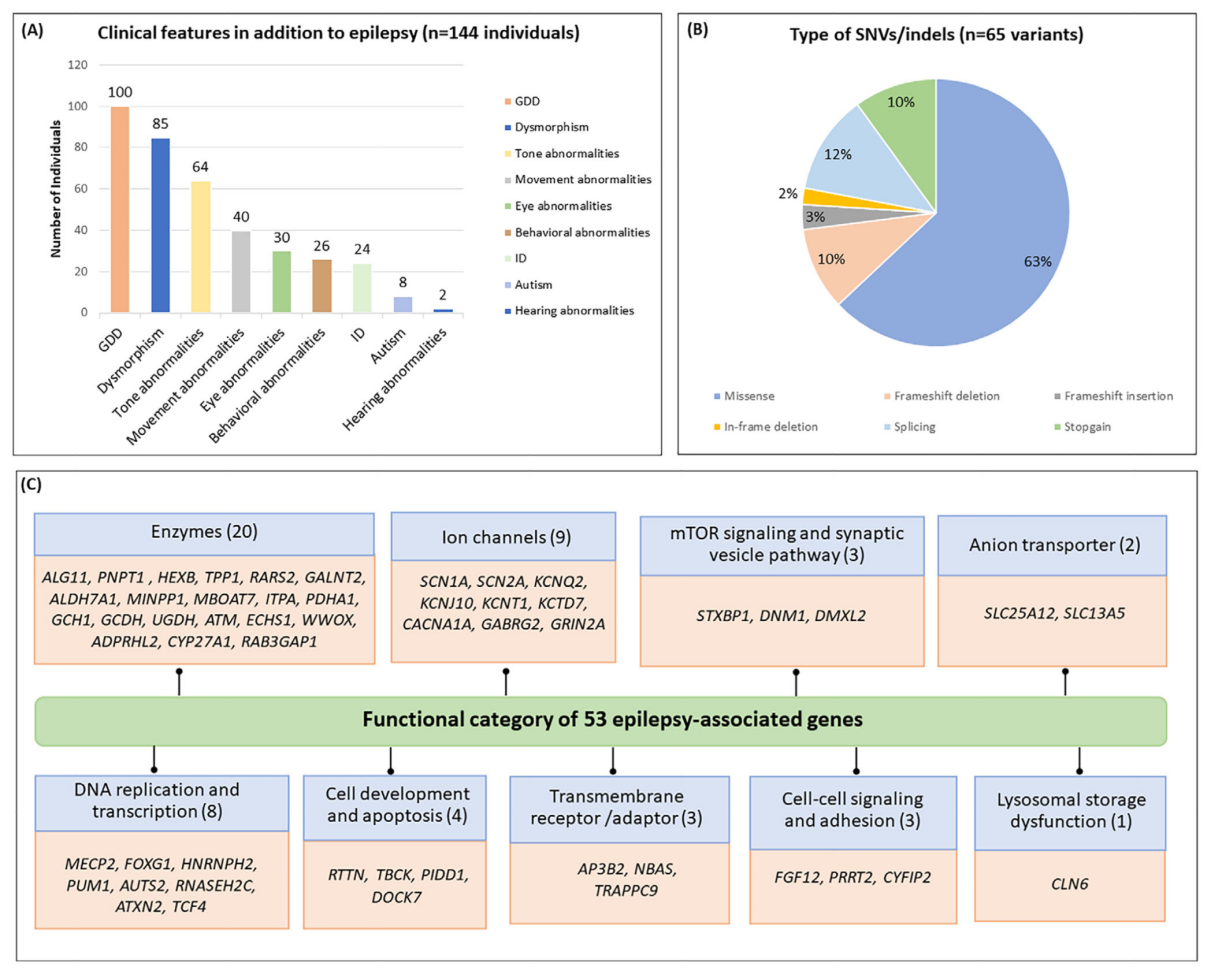
(A) Bar graph of the clinical features observed in addition to epilepsy in 144 affected individuals. ASD, autism spectrum disorder; GDD, global developmental delay; ID, intellectual disability; (B) Types of 65 single-nucleotide variants/insertion-deletions observed in the cohort (C) Schematic representation of the functional category of 53 epilepsy-associated genes observed in the cohort. [Colour figure can be viewed at wileyonlinelibrary.com]

The current cohort consists of 149 individuals from 142 families with epilepsy and their demographics, clinical, and molecular details have been shown in Table 1. Of these, 88 (59%) are males and 61 (41%) are females. Consanguinity was noted in 46 (32%) families. The age at examination ranged from the newborn period to 21 years with a median age of 3 years. The age of seizure onset ranged from day 1 to 15 years with a median age of 7 months. It was observed that 79% (118/149; 115 families) of the affected individuals had infantile-onset epilepsy (<2 years), and 21% (31/149; 27 families) had childhood-onset epilepsy (>2 years). Of these 149 individuals, isolated/familial epilepsy was observed in only five individuals (4%) while epilepsy with additional comorbidities was observed in most (144 individuals, 96%). These comorbidities included GDD, ID, tone abnormalities, movement abnormalities, behavioral abnormalities, ASD, dysmorphism, and eye abnormalities (Figure 2A).

Sixty-three of the 142 families (44%) could be classified into one of the epilepsy syndromes. Forty-eight families (76%, *n* = 63) had a diagnosis of neonatal and infantile-onset syndromes. Of these, three were defined as genetic epilepsy with febrile seizure plus (GEFS+), 24 as infantile epileptic spasm syndrome (IESS), 11 as early-infantile developmental and/or epileptic encephalopathy (EI-DEE), four as epilepsy of infancy with migrating focal seizures (EIMFS), three as Dravet syndrome, one as progressive myoclonic epilepsy (PME), and two with etiology-specific DEE. Among the 15 families with childhood onset syndromes (24%, *n* = 63), one was diagnosed with epilepsy with myoclonic-atonic seizures (EMAS), three with Lennox–Gastaut syndrome (LGS), five with EE-SWAS, and six with PME. The spectrum of epilepsy syndromes by age of onset with its corresponding molecular diagnosis is described in detail in Table 2.

A definitive molecular diagnosis was achieved in 74 of 142 families (52%) using either a single or sequential testing involving a targeted test, CMA, Mendeliome and/or ES. The details of these testing strategies and diagnostic yield are depicted in Figure 1. A total of 60 genetic disorders were identified in 74 families with epilepsy. These included 55 monogenic disorders in 66 families, four chromosomal disorders in four families, and one imprinting disorder in four families. Out of the 55 monogenic disorders, 33 were autosomal recessive (AR), 19 were autosomal dominant, and 3 were X-linked dominant disorders.

A total of 67 causative variants were identified in 66 families with monogenic disorders, of which 65 (96%) were SNVs/indels, 1 (2%) was CNV, and one (2%) was triplet-repeat expansion. The type of variants observed in the cohort have been illustrated in Figure 2B. Thirty-five of the 67 causative variants (52%) were noted to be novel. All these variants have been submitted to the ClinVar database. According to the standards and guidelines for the interpretation of sequence variants by the American College of Medical Genetics and Genomics (ACMG) and the Association for Molecular Pathology,^27^ 27 variants were pathogenic (40%), 26 were likely pathogenic (39%), and 14 were variants of uncertain significance (21%) (Table 1). Of the 66 families twith monogenic disorders, 31 had homozygous variants (47%), 6 had compound heterozygous variants (9%), 22 had heterozygous de novo variants (33%), 5 had heterozygous inherited variants (8%), and 2 had heterozygous variants of unknown inheritance (3%). Sixty-seven variants were identified in 53 epilepsy-associated genes, and the most frequently mutated genes were *PRRT2* (four families), *TPP1* (three families), *STXBP1* (three families), *SCN1A* (two families), *ALG11* (two families), *MECP2* (two families), *CLN6* (two families), *CACNA1A* (two families), and *KCNT1* (two families). Variants in remaining 44 epilepsy-associated genes were observed in one family each. These genes were further classified into 10 gene ontology categories and are represented in Figure 2C. Additional details pertaining to the clinical features, EEG findings, genetic testing performed, disease-causing variants, and ClinVar submission IDs have been provided in Tables S1 and S2.

Based on the literature, therapeutic implications were noted for 51% of families (38/74) with definitive diagnosis. Strong evidence for recommended therapies was noted in 40% (15/38) families, emerging evidence in 37% (14/38), and sparse evidence in 26% (10/38) families (Tables 3 and S1).

Among the 66 families with monogenic epilepsy, 37 families harbored variants causing autosomal recessive disorders carrying 25% risk of recurrence. Additionally, there were five families with inherited variants causing autosomal dominant disorders posing a 50% risk of recurrence in other family members. Another 22 families with de novo variants causing autosomal/X-linked dominant disorders have a negligible (<1%) risk of recurrence.

## 4 Discussion

The landscape of epilepsy genetics is broadening due to advancements in cutting-edge genomic methodologies which has led to the identification of over 800 genes associated with epilepsy within the last 20 years. This substantial discovery has significantly contributed to the extensive genetic diversity observed in epileptic disorders.^28,29^ In this study, we present a systematic analysis of 142 Indian families, revealing the phenotypic and genotypic spectrum of epilepsy and its implications for counseling and therapy.

We applied the latest ILAE 2022 diagnostic criteria for the electroclinical syndromic classification of our patient cohort. Epilepsy syndromes exhibit a wide electroclinical variability, spanning from focal epilepsy syndrome (FES) to syndromes involving DEE or with progressive neurological deterioration.^1,14^ In our study, majority (93%) of the families with the epilepsy syndrome classification fell within the DEE or neurological deterioration spectrum. Of these, IESS was most observed in 38% (24/63) of the families which is aligning with previously published cohorts with 22% and 21% of families with IESS.^8,30^ This was followed by EI-DEE in 18% (11/63), and PME in 13% (8/63) of the families in the current cohort. The ILAE 2022 classification has introduced a new category of etiology-specific syndromes which currently encompasses only six genetic conditions. However, this category is likely to expand with increasing genetic testing and consequent knowledge expansion. Hence, many of the genetic etiologies identified in the cohort which currently could not be classified into electroclinical syndromes, such as affected individuals with *KCNT1, CACNA1A, PUM1, FGF12, AP3B2, PRRT2, GRIN2A*, and Angelman syndrome may be classified as etiology-specific syndromes in the future.

Genetic disorders with epilepsy encompass a spectrum of disorders ranging from monogenic disorders, chromosomal disorders, triplet-repeat disorders, and other multifactorial disorders. The most frequently occurring monogenic disorders in pediatric onsetepilepsy cohorts include channelopathies (e.g., *SCN1A/SCN2A*-related disorder, *KCNQ2*-related disorder), followed by metabolic conditions (e.g., *SLC2A1-*related GLUT1 deficiency syndrome; *ALDH7A1*-related pyridoxine-dependent epilepsy), cell adhesion molecules (e.g., *PRRT2*-related disorder, *PCDH19*-related disorder), synaptopathies (e.g., *STXBP1*-related DEE), and mTORopathies (e.g., tuberous sclerosis due to TSC1/*TSC2* variants).^6,8,31–33^ A study by Boonsimma et al. (2022) showed that channelopathies comprised of the majority (53%) of the monogenic causes in infantile-onset epilepsies followed by neurometabolic causes (15%), and other rare genetic disorders with epilepsy (32%).^6^ In contrast, the findings of the current study show that channelopathies and metabolic disorders represent only 16% and 15% of the 55 monogenic disorders, and the majority (69%) being other rare genetic disorders with epilepsy (Table 1). The broader range of epileptic disorders observed in the present study could possibly be attributed to the prevalence of additional comorbidities in most individuals in this cohort with very few individuals manifesting isolated epilepsy. Recurrent CNVs causing well-known syndromes (17p13.3 deletion or 22q11.2 deletion syndrome) and CNVs encompassing epilepsy-associated genes in individuals with pediatric-onset epilepsy are known to contribute to the genetic etiology of disorders with epilepsy.^8,10,11^ Our results are comparable to the previous studies and we report recurrent microdeletion and imprinting disorders (Table 2). We additionally report a triplet repeat disorder in a 2-year-old female (P51) with infantile-onset spinocerebellar ataxia 2 (MIM 183090) presenting with early-onset epilepsy, facial dysmorphism, spasticity, and family history of ataxia and slurred speech. Our study, though far from being representative, highlights the clinical and genetic spectrum of epileptic disorders in the Indian population.

Though disorders with all inheritance patterns are associated with epilepsy, de novo variants causing dominant disorders constitute the most common group of genetic conditions identified in these individuals. Previous studies have shown that 50%–70% of the disease-causing variants occur de novo causing autosomal or X-linked dominant disorders.^6,11,33^ However, in the current study, it is observed that only 33% of the causative variants occurred de novo and 53% of the variants were identified in families with autosomal recessive disorders. This high rate of autosomal recessive disorders could be attributed to the prevalence of consanguinity and inbreeding in specific communities and geographic regions of India.^34,35^

The diagnostic yield of genetic testing relies significantly on factors such as age of seizure onset, presence of additional comorbidities, and the type of testing employed in individuals with epilepsy.^5,8,32,36^ The high diagnostic yield observed in our cohort can be attributed primarily to the 80% of affected individuals with seizure onset <2 years of age, and the presence of comorbidities in 94% of the individuals. Recent studies have reported cohorts of early-onset epilepsy with increased diagnostic yield ranging from 50% to 60% within the first year of life.^6,8,11^ A study by Zou et al.^11^ reported 117 of 320 affected individuals with definitive diagnosis. Of these, 74% of individuals with genetic etiology had seizure onset within the first year of life.^11^ In the current study, we found that 94% of individuals (70/74) with genetic etiology had infantile-onset epilepsy (<2 years), while 4% (4/74) had childhood-onset epilepsy (>2 years). These findings align with previously reported findings, reinforcing the association between early-onset epilepsy and a high diagnostic yield.^8,11^

We have used targeted or genomic testing first or sequential testing approach in the current study which led to an overall diagnosis of 52% in the cohort. The choice of the genetic testing employed for an individual was based on the clinical diagnosis, targeted region of interest, and the potential variant characteristics. This strategy of testing was limited to the choice of tests in order to make optimal use of the resources. We, therefore, emphasize that the clinical diagnosis and the appropriate tests employed after deep phenotyping are a rational approach for both a high yield of molecular diagnosis as well as optimal use of resources.

Advanced NGS techniques, such as exome or genome sequencing as well as epilepsy-focused gene panels have become an invaluable tool for elucidating the genetic underpinnings of epilepsies. Family-based or a trio-ES is often the preferred choice of testing for the ease of identification of de novo variants in disorders with epilepsy. Previous studies have demonstrated varied diagnostic yields for various testing modalities, that is, epilepsy-focused gene panels yielding between 15% and 47%,^32,36,37^ WES between 30% and 64%,^6,8,38,39^ and GS achieving a diagnostic rate ranging from 36% to 43%.^11,33^ In the current study, we observed a diagnostic yield of 68% and 45% using Mendeliome and ES, respectively, aligning closely with the diagnostic yields of previously reported studies. Notably, 82% of the definitive diagnosis (61/74) was achieved using proband-only Mendeliome and ES approaches, reaffirming previously reported findings observed in Indian studies that emphasize the effectiveness of robust phenotyping and singleton ES in diagnosing clinically heterogeneous genetic disorders.^31,40^ Furthermore, 93% of the definitive diagnosis (69/74) was attained using ES as a genetic test. Of note, incorporating CNV analysis algorithms from exome data has increased the diagnostic yield in identifying disease-causing variants from 49% to 52% in the current study. These results further demonstrate the effectiveness of using ES in diagnosing genetic disorders associated with epilepsy, particularly in resource-limited settings.

In resource-limited settings, access to specialized tests is limited to very few centers. However, ES has now become widely available and serves as a comprehensive and efficient tool for identifying the genetic etiology underlying disorders with epilepsy. This will pave a way for precision medicine in individuals with epilepsy. Early testing not only mitigates additional treatment expenses but also facilitates timely monitoring of epilepsy for affected individuals, ultimately enhancing their quality of life. Based on these findings, we advocate the use of ES as a first-tier test for the evaluation of diverse epilepsy cases within economically constrained regions.

Identification of precise genetic etiology has significantly advanced precision medicine in epileptic disorders. Till date, the management of epilepsies heavily relies on use of conventional AEDs. However, several recent studies have highlighted the benefits of using a targeted treatment approach based on the underlying etiology to reduce seizure frequency and improve developmental outcomes.^11,36^ In the current study, treatment implications have been noted in 51% of individuals with a definitive diagnosis, which is comparable to the previously reported studies 30%–55%.^6,33,36^ Based on this, immediate actionable treatment for biochemical disorders was available for eight families with variants in *ALDH7A1, GCDH, TPP1, GCH1*, and *PDHA1*, and AED modifications for five families with variants in *SCN1A, KCNQ2*, and *PRRT2*. We have also listed the usage or avoidance of certain AEDs for which the evidence is emerging, or sparse, and further studies or additional reports are needed to prove its efficacy (Table 2). Consequently, treatment intervention early in childhood has a great potential to improve the prognosis and developmental outcomes of the epilepsy-affected individuals. It is noteworthy that, majority of the individuals in the current cohort present with additional comorbidities which makes the treatment more challenging. Further insights into the underlying genetic mechanisms and cellular pathways in future are likely to pay the way for development of novel therapeutic strategies for management of epilepsy.

There are a few limitations of the study. The current cohort consists of majority of individuals who have epilepsy with additional comorbidities. This may have resulted in higher diagnostic yield of genetic testing when compared to the cohorts of individuals with isolated epilepsy. Despite the application of state-of-the-art genetic techniques, ~50% of the families in the current cohort remained undiagnosed. Application of trio ES, genome sequencing, long-read sequencing, and additional-omics approach can be further employed to identify other novel causative genes, non-exonic/structural variants, somatic alterations, epimutations, and digenic or oligogenic etiologies. Reanalysis of exome data too may increase the diagnostic yield based on the updated new information in the literature. In addition, though the study highlights the implications of genetic testing on treatment, it lacks longitudinal clinical outcomes, hindering insights into precision medicine’s efficacy for epilepsy.

In conclusion, the spectrum of epileptic disorders is expanding with a rapid rate driven by technological advances and gene discovery. Therefore, the integration of early genetic testing and deep phenotyping of individuals with epilepsy in healthcare settings will help establish accurate diagnosis and has potential benefits in terms of reducing the economic burden by preventing additional investigations. We further demonstrate the feasibility of using singleton ES as a first-tier test in a resource-limited setting to facilitate early genetic diagnosis, genetic counseling, and precision medicine. Comprehensive and systematic studies across diverse populations are likely to contribute to the increasing pool of disease-causing variants and epileptic disorders globally which will further help us understand the complete genetic landscape of epilepsy.

## Supplementary Material

Supplementary file

Supplementary tables

## Figures and Tables

**Table 1:** Cohort characteristics of families with epilepsy.

Clinical characteristics	Number (Percentage)
**Demographics**	
** Total number of individuals**	149
Male	88 (59%)
Female	61 (41%)
** Total number of families**	142
Consanguinity	46 (32%)
Non-consanguinity	96 (68%)
** Age range at examination**	Birth to 21 years
** Median age at examination**	3 years
**Age of seizure onset**	
Infantile-onset (<2 years)	118 (79%) individuals
Childhood-onset (>2 years)	31 (21%) individuals
**Clinical features**	
Isolated epilepsy	5 (4%) individuals
Additional comorbidities	144 (96%) individuals
**Epilepsy syndrome**	
** Neonatal and infantile-onset**	**48 (76%) families**
GEFS+	3(6%)
IESS	24 (50%)
EI-DEE	11 (23%)
EIMFS	4(9%)
DS	3 (6%)
PME	1 (2%)
Etiology-specific DEE	2 (4%)
** Childhood-onset**	**15 (24%) families**
EMAS	1 (7%)
LGS	3 (20%)
EE-SWAS	5 (33%)
PME	6 (40%)
**Genetic tests**	
** Molecular diagnosis**	**74 (52%) families**
Targeted test	4 families
CMA	1 family
Mendeliome	12 families
Singleton ES	52 families
Trio ES	5 families
**Disorders**	
** Monogenic disorders**	66 (90%) families
Autosomal recessive	37 (56%) families
Autosomal dominant	25 (38%) families
X-linked dominant	4 (6%) families
** Chromosomal disorders**	4 (5%) families
** Imprinting disorders**	4 (5%) families
**Variants**	67
Known	32 (48%)
Novel	35 (52%)
Pathogenic	27 (40%)
Likely pathogenic	26 (39%)
Variants of uncertain significance	14 (21%)
**Clinical implications**	
** High risk of recurrence**	42 (57%) families
** Therapeutic implications**	38 (51%) families

Abbreviations: CMA, chromosomal microarray; DS, Dravet syndrome; EE-SWAS, epileptic encephalopathy with spike-and-wave activation in sleep; EIDEE, early-infantile developmental and/or epileptic encephalopathy; EIMFS, epilepsy of infancy with migrating focal seizures; EMAS, epilepsy with myoclonic-atonic seizures; ES, exome sequencing; GEFS+, genetic epilepsy with febrile seizure plus; IESS, infantile epileptic spasm syndrome; LGS, Lennox–Gastaut syndrome; PME, progressive myoclonic epilepsy.

**Table 2:** Epilepsy syndrome classification and molecular diagnosis observed in the cohort (*n* = 142 families).

Epilepsy syndrome (total number of families)	Diagnosed families (%)		Proband ID		Age/gender	Final diagnosis (MIM)	Inheritance pattern	Gene	Variant nomenclature	Zygosity (inheritance)	ACMG classification
**Onset in infants and neonates (<2 years)**											
**Focal/generalized syndromes**											
Genetic epilepsy with febrile seizures plus (3)	-		-		-	-	-	-	-	-	-
**Syndromes with DEE or neurological deterioration**											
Infantile epileptic spasm syndrome (24)	11/24 (44%)		Pl		6 months/M	Congenital disorder of glycosylation, type Ip (613661)	AR	*ALG11*	NM_001004127.3: c.1241T > A p.(Ile414Asn)	Homozygous (maternal and paternal)	VUS
			P2		9 months/M	Congenital disorder of glycosylation, type Ip (613661)	AR	*ALG11*	NM_001004127.3: c.1241T > A p.(Ile414Asn)	Homozygous (maternal and paternal)	VUS
			P3		5 Y/F	Combined oxidative phosphorylation deficiency 13 (614932)	AR	*PNPT1*	NM_033109.5:c.2213G > A p. (Arg738His)^a^	Homozygous (maternal and paternal)	Likely pathogenic
			P24		3 Y/M	Microcephaly, short stature, and polymicrogyria with seizures (614833)	AR	*RTTN*	NM_173630.4:c.4438C > T p. (Leul480Phe)^a^	Homozygous (maternal and paternal)	VUS
			P31		8 Y/F	Intellectual developmental disorder, autosomal recessive 57 (617188)	AR	*MBOAT7*	NM_024298.5:c.1290C > A p. (Tyr430Ter)^a^	Homozygous (maternal and paternal)	VUS
			P49		2 Y/F	Pyruvate dehydrogenase El-alpha deficiency (312170)	XLD	*PDHA1*	NM_000284.4:c.379C > T p. (Argl27Trp)	Heterozygous (de novo)	Pathogenic
			P64		2 Y/F	Developmental and epileptic encephalopathy 28 (616211)	AR	*WWOX*	NM_016373.4:c.172 + 5G > A^a^	Homozygous (maternal and paternal)	VUS
			P51		2 Y/F	Spinocerebellar ataxia 2 (183090)	AD	*ATXN2*	Heterozygous for CAG repat expansion at SCA2 locus (20/13 repeat size)	Heterozygous (paternally inherited)	Pathogenic
			P70		1Y/M	Cerebrotendinous xanthomatosis (213700)	AR	*CYP27A1*	NM_000784.4: c.490C > T p. (Arg164Trp)^a^; c.1184 + 1G > A	Compound heterozygous^b^	Likely pathogenic/ Pathogenic
			P29		19 months/M	15ql3.3 microdeletion syndrome	AD	NA	arr[GRCh38] 15q13.2q13.3 (30621371_32622888) x1 (2Mb deletion)	Heterozygous deletion	Pathogenic
Early infantile DEE (11)	7/11 (64%)		P8		5 Y/F	Developmental and epileptic encephalopathy 4 (612164)	AD	*STXBP1*	NM_001032221.6: c.785A > T p.(Asp262Val)^a^	Heterozygous (de novo)	Likely pathogenic
			P10		2 Y/M	Developmental and epileptic encephalopathy 4 (612164)	AD	*STXBP1*	NM_001032221.6: c.1217G > A p.(Arg406His)	Heterozygous (de novo)	Likely pathogenic
			P25		2 Y/M	Developmental and epileptic encephalopathy 4 (612164)	AD	STXBP1	NM_001032221.6: c.1359 + 5G > A^a^	Heterozygous (de novo)	VUS
			P15		4 months/F	Developmental and epileptic encephalopathy 65 (618008)	AD	*CYFIP2*	NM_001037333.3: c.2089 T > C p.(Cys697Arg)^a^	Heterozygous (de novo)	Likely pathogenic
			P23		1 Y/M	Developmental and epileptic encephalopathy 25, with amelogenesis imperfecta (615905)	AR	*SLC13A5*	NM_177550.5:c.659G > A p. (Gly220Asp)^a^	Homozygous (maternal and paternal)	VUS
			P28		1 month/M	Pontocerebellar hypoplasia, type 16 (619527)	AR	M/NPP1	NM_004897.5:c.1008C > G p. (Ser336Arg)^a^	Homozygous (maternal and paternal)	VUS
			P36		3 months/M	Developmental and epileptic encephalopathy 81 (618663)	AR	*DMXL2*	NM_001378457.1:c.352 T > C p.(Trp118Arg)^a^	Homozygous (maternal and paternal)	VUS
Epilepsy of infancy with migrating focal seizures (4)	4/4 (100%)		P20		3 Y/M	Developmental and epileptic encephalopathy 14 (614959)	AD	*KCNT1*	NM_020822.3:c.3001A > T p. (Thr1001Ser)^a^	Heterozygous (de novo)	Likely pathogenic
			P21		1 month/F	Developmental and epileptic encephalopathy 14 (614959)	AD	*KCNT1*	NM_020822.3:c.1546A > G p. (Met516Val)	Heterozygous (de novo)	Pathogenic
			P34		4 Y/M	Developmental and epileptic encephalopathy 31 (616346)	AD	*DNM1*	NM_001288739.2: c.1197-8G > A	Heterozygous (de novo)	Likely pathogenic
			P45		5 months/F	Developmental and epileptic encephalopathy 35 (616647)	AR	*ITPA*	NM_033453.4:c.137delA p. (Gln46Argfs*43)^a^	Homozygous (maternal and paternal)	Pathogenic
Dravet syndrome (3)	3/4 (75%)		P6		3 Y/F	Epilepsy, generalized, with febrile seizures plus, type 2 (604403)	AD	*SCN1A*	NM_006920.6:c.5087 T > C p. (Phel696Ser)^a^	Heterozygous (de novo)	Pathogenic
			P77		1 Y/F	Developmental and epileptic encephalopathy 6B, non- Dravet (619317)	AD	*SCN1A*	NM_001165963.4: c.677C > T p.(Thr226Met)	Heterozygous (de novo)	Pathogenic
			P33		3 Y/M	SCN2A-related disorder	AD	*SCN2A*	NM_001040142.2: c.2765G > A p.(Arg922His)^a^	Heterozygous (de novo)	Likely pathogenic
Progressive myoclonic epilepsy (1)	1/1 (100%)		P76		2 Y/M	Epilepsy, progressive myoclonic 3, with or without intracellular inclusions (611726)	AR	*KCTD7*	NM_153033.5: c.518 T > A p. (llel73Asn)^a^	Homozygous (maternal and paternal)	VUS
Etiology-specific DEEs (2)	(2/2,100%)		P26		Day 12/M	Epilepsy, pyridoxine-dependent (266100) (1, 100%)	AR	*ALDH7A1*	NM_001182.5: c.1003C > T p. (Arg335Ter)/ c,1411_1412insG p. (Leu471ArgfsTer4)^a^	Compound heterozygous (maternal and paternal)	Pathogenic
			P81		9Y/M	*KCNQ2*-related disorder	AD	*KCNQ2*	NM_172107.4: c.833 T > C p. (lle278Thr)	Heterozygous (de novo)	Likely pathogenic
Non-syndromic epilepsy (64)	31/64 (48%)		P13		1 Y/F	Developmental and epileptic encephalopathy 48 (617276)	AD	*AP3B2*	NM_001278512.2: c.2978_2979del p. (Pro993ArgfsTer5)	Heterozygous (de novo)	Pathogenic
			P14		2 Y/M	Developmental and epileptic encephalopathy 47 (617166)	AD	*FGF12*	NM_021032.5:c.334G > A p. (Gly112Ser)	Heterozygous (de novo)	Likely pathogenic
			P16, P17^c^		5 Y/F	Congenital disorder of glycosylation, type lit (618885)	AR	*GALNT2*	NM_004481.5:c.623G > A p. (Arg208Gln)^a^	Homozygous (maternal and paternal)	Likely pathogenic
			P30		1 Y/M	Developmental and epileptic encephalopathy 39 (612949)	AR	*SLC25A12*	NM_003705.5:c.1469G > A p. (Arg490Gln)	Homozygous (maternal and paternal)	VUS
			P32		1 Y/M	Rett syndrome, congenital variant (613454)	AD	*FOXG1*	NM_005249.5: c.312_333del p.(Prol05ArgfsTer80)^a^	Heterozygous (de novo)	Pathogenic
			P35		2 Y/F	Angelman syndrome	NA	*NA*	Heterozygous deletion in PWS/AS region (15q11)	Heterozygous (NA)	Pathogenic
			P37		4 Y/F	Rett syndrome (312750)	XLD	*MECP2*	NM_001110792.2:c.842delG p.(Gly281AlafsTer20)	Heterozygous (de novo)	Pathogenic
			P38		7 Y/F	Angelman syndrome	NA	NA	arr[GRCh38] 15q11.2ql3.3 (22582283_32623522)xl (10.04 Mb deletion)	Heterozygous	Pathogenic
			P40		1 Y/M	Developmental and epileptic encephalopathy 42 (617106)	AD	*CACNA1A*	NM_001127222.2: c.4043G > A p.(Argl348Gln)	Heterozygous (de novo)	Likely pathogenic
			P41		1 Y/F	Developmental and epileptic encephalopathy 42 (617106)	AD	*CACNA1A*	NM_001127222.2: c.4043G > A p.(Argl348Gln)	Heterozygous^b^	Likely pathogenic
			P44		13 Y/M	22q11.2 microdeletion syndrome	AD	NA	arr[GRCh38]22q11.21 (18166089_21110475)x1 (2.9 Mb deletion)	Heterozygous	Pathogenic
			P46		9 Y/F	Short stature, optic nerve atrophy, and Pelger-Huet anomaly	AR	*NBAS*	NM_015909.4:c.886-3C > G^a^	Homozygous (maternal and paternal)	VUS
			P47		4 Y/F	Intellectual developmental disorder, autosomal recessive 13 (613192)	AR	*TRAPPC9*	NM_001160372.4:c.2699 + 1G > A^a^	Homozygous (maternal and paternal)	Pathogenic
			P50		2 Y/F	Hyperphenylalaninemia, BH4-deficient, B (233910)	AR	*GCH1*	NM_000161.3:c.644 T > C p. (Met215Thr)^a^	Homozygous (maternal and paternal)	Likely pathogenic
			P52		1 Y/M	Spinocerebellar ataxia 47	AD	*PUM1*	NM_001020658.2: c.3439C > T p.(Arg1147Trp)	Heterozygous (de novo)	Likely pathogenic
			P53		2 Y/F	Glutaric Acidemia Type 1 (231670)	AR	*GCDH*	NM_000159.4:c.281G > A p. (Arg94Gln); c.1204C > T p. (Arg402Trp)	Compound heterozygous (maternal and paternal)	Pathogenic/ Pathogenic
			P63		1 Y/M	Angelman syndrome	NA	NA	Heterozygous deletion in PWS/AS region (15q11)	Heterozygous (NA)	Pathogenic
			P65		3 Y/M	Neurodegeneration, childhood-onset, stress-induced, with variable ataxia and seizures (618170)	AR	*ADPRHL2*	NM_017825.3:c.414_418del p. (Alal39GlyfsTer4)	Homozygous (maternal and paternal)	VUS
			P42		10 months/M	*PRRT2*-related disorder	AD	*PRRT2*	NM_145239.3:c.649dup p. (Arg217ProfsTer8)	Heterozygous (maternal)	Pathogenic
			P56		1 Y/M	Aicardi-Goutieres syndrome 3 (610329)	AR	*RNASEH2C*	NM_032193.4: c.205C > T p. (Arg69Trp)	Homozygous (maternal and paternal)	Likely pathogenic
			P57		5 Y/F	Ataxia-telangiectasia (208900)	AR	*ATM*	NM_000051.4: c.4852C > T p. (Argl618Ter)	Homozygous (maternal and paternal)	Pathogenic
			P58, P59^c^		17Y/F, 9 Y/M	Hypotonia, infantile, with psychomotor retardation and characteristic facies 3, (616900)	AR	*TBCK*	NM_001163435.3: c.557A > G Ñ€. (Aspl86Gly)^a^/ Ñ?. 737_738del (p.Val246AspfsTer6)^a^	Compound heterozygous (maternal and paternal)	Likely pathogenic/ Pathogenic
			P60		4 Y/F	1q43q44 microdeletion syndrome	AD	NA	arr[GRCh38] 1q43q44 (238726812_246194815)x1 (7.4 Mb)	Heterozygous	Pathogenic
			P61		1 Y/F	Developmental and epileptic encephalopathy 74 (618396)	AD	*GABRG2*	NM_198904.4:c.853C > G p. (Leu285Val)^a^	Heterozygous (de novo)	Likely pathogenic
			P62		8 months/M	Mitochondrial short-chain enoyl-CoA hydratase 1 deficiency (616277)	AR	*ECHS1*	NM_004092.4:c.476A > G p. (Gln159Arg)	Homozygous	Likely pathogenic
			P75		3 Y/M	Intellectual developmental disorder 75, with neuropsychiatric features and variant lissencephaly (619827)	AR	*PIDD1*	NM_145886.4:c.2134C > T p. (Arg712Trp)^a^; c.2507 T > C p.(Leu836Pro)^a^	Compound heterozygous (maternal and Paternal)	VUS
			P78		1 Y/F	Developmental and epileptic encephalopathy, 23 (615859)	AR	*DOCK7*	NM_001367561.1: c.2112 + 2 T > C^a^	Homozygous (maternal and paternal)	Pathogenic
			P68		13 Y/M	PRRT2-related disorder	AD	*PRRT2*	NM_145239.3:c.649dup p. (Arg217ProfsTer8)	Heterozygous (paternal)	Pathogenic
			P80		9 Y/M	PRRT2-related disorder	AR	PRRT2	NM_145239.3:c.649dup p. (Arg217ProfsTer8)	Homozygous (maternal and paternal)	Pathogenic
			P69		1 Y/M	Warburg micro syndrome 1 (600118)	AR	*RAB3GAP1*	NM_012233.3: c.2290-17_2290del	Homozygous (maternal)^b^	Likely pathogenic
			P19		1 Y/M	PRRT2-related disorder	AD	*PRRT2*	NM_145239.3:c.649dup p. (Arg217ProfsTer8)	Heterozygous (paternal)	Pathogenic
Onset in childhood (>2 years)											
**Syndromes with DEE or neurological deterioration**											
Epilepsy with myoclonic-atonic seizures (1)	0/1 (0%)		-		-	-	-	*-*	-	-	-
Lennox-Gastaut syndrome (3)	1/3 (33%)		P5		4 Y/F	Rett syndrome (312750) (1, 50%)	XLD	*MECP2*	Exon 4 deletion	Heterozygous	Pathogenic
Epileptic encephalopathy with spike- and-wave activation in sleep (5)	2/5 (40%)		P18 P79		3 Y/M 11 Y/M	SESAME syndrome (612780) (1, 50%) Intellectual developmental disorder, autosomal dominant 26 (615834)	AR AD	*KCNJ10* *AUTS2*	NM_002241.5:c.76C > T p. (Arg26Ter) NM_015570.4:c.742_742 + 3del^a^	Homozygous (maternal and paternal) Heterozygous (de novo)	Pathogenic Pathogenic
			P79		11 Y/M	Intellectual developmental disorder, autosomal dominant 26 (615834)	AD	*AUTS2*	NM_015570.4:c.742_742 + 3del^a^	Heterozygous (de novo)	Pathogenic
Progressive myoclonic epilepsy (6)	4/6 (67%)		P7		5 Y/M	Ceroid lipofuscinosis, neuronal, 6 (601780)	AR	*CLN6*	NM_017882.3:c.896C > A p. (Pro299His)^a^	Homozygous (maternal and paternal)	Likely pathogenic
			P12		8 Y/F	Pontocerebellar hypoplasia, type 6 (611523)	AR	*RARS2*	NM_020320.5:c.848 T > A p. (Leu283Gln)	Homozygous (maternal and paternal)	Likely pathogenic
			P71		5 Y/M	Ceroid lipofuscinosis, neuronal, 2 (204500)	AR	*TPP1*	NM_000391.4: c.622C > T p. (Arg208Ter)	Homozygous (maternal and paternal)	Pathogenic
			P22		4 Y/M	Ceroid lipofuscinosis, neuronal, 6 (601780)	AR	*CLN6*	NM_017882.3:c.794_796del p. (Ser265del)	Homozygous (maternal and paternal)	Likely pathogenic
Non-syndromic epilepsy (13)	6/13 (50%)			P4	7 Y/M	Sandhoff disease, infantile, juvenile forms (268800)	AR	*HEXB*	NM_000521.4:c.965 T > C p. (lle322Thr)^a^	Homozygous (maternal and paternal)	VUS
				P9	5 Y/F	Ceroid lipofuscinosis, neuronal, 2 (204500)	AR	*TPP1*	NM_000391.4:c.688-1G > T^a^; c.1340G > C p.(Arg447Pro)^a^	Compound heterozygous (maternal and paternal)	Pathogenic/ Likely pathogenic
				P11	4 Y/F	Ceroid lipofuscinosis, neuronal, 2 (204500)	AR	*TPP1*	NM_000391.4:c.379C > T p. (Arg127Ter)	Homozygous (maternal and paternal)	Pathogenic
				P67	6 Y/F	Pitt-Hopkins syndrome (610954)	AD	*TCF4*	NM_001083962.2: c.655G > C p.(Asp219His)^a^	Heterozygous (de novo)	Likely pathogenic
				P72, P73^c^	8 Y/F, 8 Y/F	Epilepsy, focal, with speech disorder and with or without impaired intellectual development (245570)	AD	*GR/N2A*	NM_001134407.3: c.2179G > A p.(Ala727Thr)	Heterozygous (maternal)	Pathogenic
				P39	5 Y/F	Intellectual developmental disorder, X-linked syndromic, Bain type (300986)	XLD	*HNRNPH2*	NM_019597.5:c.629A > G p. (Tyr210Cys)	Heterozygous (de novo)	Likely pathogenic

Abbreviations: AD, autosomal dominant; AR, autosomal recessive; DEE, developmental and/or epileptic encephalopathy; F, Female; ID, identifier; M, Male; NA, not applicable; VUS, variant of uncertain significance; XLD, X-linked dominant; Y, years.

a Novel variants not reported in the literature.

b Paternal sample is not available for segregation.

c Probands from one family.

**Table 3:** Gene-specific therapeutic implications observed in the cohort.

Proband ID	Gene	Anti-epileptic drugs		Other therapeutic implications	Strength of evidence
		Indicated	Contraindicated		
P53	*GCDH*	-	-	Low lysine & tryptophan diet, carnitine supplementation	Strong
P11/P9/P71	*TPP1*	-	-	Tripeptidyl-peptidase I enzyme replacement therapy	Strong
P26	*ALDH7A1*	-	-	Pyridoxine and folinic acid	Strong
P49	*PDHA1*	Phenytoin, clobazam	-	Ketogenic diet	Strong
P6/P77	*SCN1A*	Stiripentol, sodium valproate, clobazam, benzodiazepines, cannabinoids	Sodium channel blockers (carbamazepine, oxcarbazepine, phenytoin and lamotrigine)	Ketogenic diet	Strong
P50	*GCH1*	-	-	L-DOPA, 5-hydroxytryptophan, sapropterin	Strong
P81	*KCNQ2*	Phenytoin, carbamazepine, retigabine	-	-	Strong
P68/P80/P19/P42	*PRRT2*	Carbamazepine, oxcarbazepine, sodium valproate, phenobarbital	-	Avoiding stress, sleep deprivation, anxiety, and other triggers	Strong
P33	*SCN2A*	Valproic acid, benzodiazepine, levetiracetam, oxcarbazepine	Sodium channel blockers	-	Strong (for onset of >3 months of age)
P8/P10/P25	*STXBP1*	Vigabatrin, sodium valproate, levetiracetam, ACTH	-	-	Emerging
P20/P21	*KCNT1*	Quinidine	-	-	Emerging
P40/P41	*CACNA1A*	Acetazolamide, calcium channel blockers	-	-	Emerging
P5/P37	*MECP2*	Valproic acid, carbamazepine, lamotrigine		Cannabidivarin	Emerging
P30	*SLC25A12*	Levetiracetam, phenobarbital		Ketogenic diet	Emerging
P23	*SLC13A5*	Sodium valproate, acetazolamide, carbamazepine, stiripentol	-	-	Emerging
P72	*GRIN2A*	Memantine (GoF variants), L-Serine (LoF variants)	-	-	Emerging
P7/P22	*CLN6*	Sodium valproate, Lamotrigine	Phenytoin, carbamazepine	-	Emerging
P34	*DNM1*	-	-	Ketogenic diet	Sparse
P1/P2	*ALG11*	Topiramate, vigabatrin		Ketogenic diet	Sparse
P18	*KCNJ10*	Sodium valproate	-	-	Sparse
P12	*RARS2*	Sodium valproate, levetiracetam, topiramate, Clonazepam	-	-	Sparse
P13	*AP3B2*	Vigabatrin	-	-	Sparse
P14	*FGF12*	Sodium valproate, topiramate, carbamazepine, clobazam, levetiracetam, phenytoin	-	-	Sparse
P61	*GABRG2*	Sodium valproate and levetiracetam	-	-	Sparse
P3	*PNPT1*	Phenobarbitone, vigabatrin, sodium valproate	-	-	Sparse
P55	*UGDH*	Sodium valproate	-	Ketogenic diet	Sparse

Abbreviations: ACTH, adrenocorticotropic hormone; GoF, gain-of-function variants; LoF, loss-of-function variants.

## Data Availability

The data that support the findings of this study are available from the corresponding author upon reasonable request.
