# Supplementary material for: Genetic and phenotypic landscape of pediatric-onset epilepsy in 142 Indian families: Counseling and therapeutic implications
Source: Clin Genet. Author manuscript; Available in PMC 2024 Jun 1. (PMC7615923; doi:10.1111/cge.14495)
Supplement: Supplementary file [file EMS195231-supplement-Supplementary_file.docx]

**Genetic and phenotypic landscape of pediatric-onset epilepsy in 142 Indian families: Counselling and therapeutic implications**

**Supplementary material**

***Detailed methodology***

***DNA extraction***

2-5 ml EDTA blood was collected from the proband and their respective parents as available. The genomic DNA was extracted using QIAmp DNA Blood Mini kit and QIAmp DNA Blood Mini kit (QIAGEN, Hilden, Germany) following the standard protocols.

***Chromosomal microarray and analysis of copy number variants***

CMA was performed using the Illumina’s Infinium Global Screening Array (650k array, San Diego, California) and Affymetrix CytoScan 750K array (Santa Clara, California). The resulting data was analysed using Chromosome Analysis Suite (ChAS) v.4.2.1, and KaryoStudio v1.4. The details are provided in supplementary table S5.

Publicly available database like the Database of Genomic variants (DGV) and Database of chromosomal imbalances and phenotypes in human using ensemble resources (DECIPHER), gnomAD, ClinVar and the published literature were used to study the CNVs identified in the affected individuals. Briefly, the CNVs were evaluated for being rare or commonly reported in the population; presence of protein coding or morbid genes in the CNV interval, co-relation with patient phenotypes, the disease mechanism and inheritance pattern. The significance of the identified CNVs in an individual were then classified into pathogenic, likely pathogenic, variant of uncertain significance, likely benign or benign based on American College of Medical Genetics and Genomics (ACMG) guidelines and ClinGen criteria.

**Mendeliome**

Based upon the provisional clinical diagnosis of a known monogenic disorder, a Mendeliome or targeted exome sequencing was performed in five families with achievement of a molecular diagnosis in all the families. A Mendeliome involved next generation sequencing of exons of 6670 genes using the Clinical Exome version 4 capture kit (MedGenome Labs. Pvt Ltd.). The processing, annotating, filtering and analyzing strategies were same as that for exome sequencing as described above.

**Exome sequencing**

Exome sequencing (ES) was performed using standard protocols (1). The NGS platforms, capture kits and read depth details used for exome sequencing in the cohort are given in supplementary table S4. The first step was filtering the variants with minor allele frequency of <2% in population database like gnomAD and ExAC (2,3) and our in-house data of 2978 exomes comprised of affected individuals with rare mendelian disorders and their unaffected parents (4). The next step was prioritization based upon the location of the variant. Intergenic, and deep intronic variants are excluded as it is believed that at least 80% of the disease-causing variants underlying the monogenic disorder lie in the protein coding or the exonic region of the genome (5). Variants were then segregated based on zygosity (homozygous, heterozygous, compound heterozygous and hemizygous). The remaining variants were prioritized based on concordance with phenotype observed in the patient with Online Mendelian Inheritance in Man (OMIM) phenotypes and Human Phenotype Ontology (HPO) terms and multiple lines of *in silico* pathogenicity predictions. Sanger sequencing was performed as necessary either for segregation analysis or for validating the same.

**Copy number variant analysis from exome sequencing data**

CNV analysis from the exome sequencing data was carried out in patients where exome analysis did not reveal any significant variant. Briefly, “eXome-Hidden Markov Model (XHMM)”, “ExomeDepth” and “cn.MOPS” protocol were followed for CNV calling from the binary alignment file (6-8) . CNVs were called against ES data of capture-kit matched reference samples from our in-house dataset (CREv2, CREv3, CREv3, CEV4 and TWIST). CNVs in genomic regions/genes previously implicated in a specific multiple congenital anomaly syndrome or disease were filtered for any significant variations along with its rarity. The retrieved CNV’s were then filtered using Database of Genomic Variants (DGV) and gnomAD or Database of Chromosomal Imbalance and Phenotype in Humans Using Ensemble Resources (DECIPHER) and ClinVar to elucidate its presence in population or in affected individuals respectively. The CNV interval was looked for significant genes for concordance with inheritance pattern, disease mechanism, phenotypes associated from the literature in addition to referring to the currently evolving CNV interpretation tools like ClinGen (9). The called CNVs were then validated using either chromosomal microarray or MS-MLPA.

**Table S4.** NGS platforms, capture kit and read depth details used for exome sequencing in the cohort.

| **Platform** | **Type of**  **reads** | **Capture kit** | **Target**  **region** | **Sequencing**  **coverage at 10X** |
| --- | --- | --- | --- | --- |
| NovaSeq6000 | PE 2x150 | Agilent SureSelect Clinical Research Exome v2 (CREv2, Santa Clara, California) | 67mb | >95% |
| NovaSeq6000 | PE 2x150 | Agilent SureSelect Clinical Research Exome v3 (CREv3, Santa Clara, California) | 70mb | >95% |
| NovaSeq6000 | PE 2x150 | Agilent SureSelect Clinical Research Exome v4 (CREv4, Santa Clara, California) | 41.1mb | >95% |
| NovaSeq6000 | PE 2x150 | Clinical Exome version 4 | 50mb | >95% |
| NovaSeq6000 | PE 2x150 | TWIST and a modified TWIST (South San Francisco California) | 50mb | >95% |

**Supplementary table S5:** Chromosomal microarray platforms and analysis software

| **S no** | **Microarray platform** | **Analysis software** |
| --- | --- | --- |
| 1 | Affymetrix CytoScan 750K array (Santa Clara, California) | Chromosome Analysis Suite (ChAS) v4.2 |
| 2 | Illumina’s Infinium Global Screening Array BeadChip (San Diego, California) | KaryoStudio v1.4; Genomestudio v2.0 |

**References:**

1. Kaur P, do Rosario MC, Hebbar M, Sharma S, Kausthubham N, Nair K, et al. Clinical and genetic spectrum of 104 Indian families with central nervous system white matter abnormalities. Clin Genet. 2021;100(5):542-50.
2. Lek M, Karczewski KJ, Minikel EV, Samocha KE, Banks E, Fennell T, et al. Analysis of protein-coding genetic variation in 60,706 humans. Nature. 2016;536(7616):285-91.
3. Karczewski KJ, Weisburd B, Thomas B, et al. The ExAC browser: displaying reference data information from over 60 000 exomes. *Nucleic Acids Res*. 2017;45(D1):D840-D845.
4. Kausthubham N, Shukla A, Gupta N, Bhavani GS, Kulshrestha S, Das Bhowmik A, et al. A data set of variants derived from 1455 clinical and research exomes is efficient in variant prioritization for early-onset monogenic disorders in Indians. Hum Mutat. 2021;42(4):e15-e61.
5. Lupski JR, Belmont JW, Boerwinkle E, Gibbs RA. Clan genomics and the complex architecture of human disease. Cell. 2011;147(1):32-43.
6. Fromer M, Moran JL, Chambert K, et al. Discovery and statistical genotyping of copy-number variation from whole-exome sequencing depth. *Am J Hum Genet*. 2012;91(4):597-607.
7. Plagnol V, Curtis J, Epstein M, et al. A robust model for read count data in exome sequencing experiments and implications for copy number variant calling. *Bioinformatics*. 2012;28(21):2747-2754.
8. Klambauer G, Schwarzbauer K, Mayr A, et al. cn.MOPS: mixture of Poissons for discovering copy number variations in next-generation sequencing data with a low false discovery rate. *Nucleic Acids Res*. 2012;40(9):e69.
9. Rehm HL, Berg JS, Brooks LD, Bustamante CD, Evans JP, Landrum MJ, et al. ClinGen--the Clinical Genome Resource. N Engl J Med. 2015;372(23):2235-42.
